# Molecular Characterization of the Convergent Carbapenem-Resistant and Hypervirulent Klebsiella pneumoniae Strain K1-ST23, Collected in Chile during the COVID-19 Pandemic

**DOI:** 10.1128/spectrum.00540-23

**Published:** 2023-05-16

**Authors:** Felipe Morales-León, Maximiliano Matus-Köhler, Pablo Araya-Vega, Felipe Aguilera, Ignacio Torres, Rodrigo Vera, Camila Ibarra, Sebastián Venegas, Helia Bello-Toledo, Gerardo González-Rocha, Andrés Opazo-Capurro

**Affiliations:** a Laboratorio de Investigación en Agentes Antibacterianos, Departamento de Microbiología, Facultad de Ciencias Biológicas, Universidad de Concepción, Concepción, Chile; b Departamento de Farmacia, Facultad de Farmacia, Universidad de Concepción, Concepción, Chile; c Departamento de Bioquímica y Biología Molecular, Facultad de Ciencias Biológicas, Universidad de Concepción, Concepción, Chile; d Hospital de Urgencia Asistencia Pública, Santiago de Chile, Chile; Universidade de Sao Paulo

**Keywords:** carbapenem-resistant *Klebsiella pneumoniae*, WHO priority pathogens, hypervirulence, genomic surveillance, carbapenem resistance

## Abstract

The aim of this study was to investigate the genomic features of a carbapenem-resistant hypervirulent Klebsiella pneumoniae (CR-hvKp) isolate (K-2157) collected in Chile. Antibiotic susceptibility was determined using the disk diffusion and broth microdilution methods. Whole-genome sequencing (WGS) and hybrid assembly were performed, using data generated on the Illumina and Nanopore platforms. The mucoid phenotype was analyzed using both the string test and sedimentation profile. The genomic features of K-2157 (e.g., sequence type, K locus, and mobile genetic elements) were retrieved using different bioinformatic tools. Strain K-2157 exhibited resistance to carbapenems and was identified as a high-risk virulent clone belonging to capsular serotype K1 and sequence type 23 (ST23). Strikingly, K-2157 displayed a resistome composed of β-lactam resistance genes (*bla*_SHV-190_, *bla*_TEM-1_, *bla*_OXA-9_, and *bla*_KPC-2_), the fosfomycin resistance gene *fosA*, and the fluoroquinolones resistance genes *oqxA* and *oqxB*. Moreover, several genes involved in siderophore biosynthesis (*ybt*, *iro*, and *iuc*), bacteriocins (*clb*), and capsule hyperproduction (plasmid-borne *rmpA* [_p_*rmpA*] and _p_*rmpA2*) were found, which is congruent with the positive string test displayed by K-2157. In addition, K-2157 harbored two plasmids: one of 113,644 bp (KPC+) and another of 230,602 bp, containing virulence genes, in addition to an integrative and conjugative element (ICE) embedded on its chromosome, revealing that the presence of these mobile genetic elements mediates the convergence between virulence and antibiotic resistance. Our report is the first genomic characterization of a hypervirulent and highly resistant K. pneumoniae isolate in Chile, which was collected during the coronavirus disease 2019 (COVID-19) pandemic. Due to their global dissemination and public health impact, genomic surveillance of the spread of convergent high-risk K1-ST23 K. pneumoniae clones should be highly prioritized.

**IMPORTANCE** Klebsiella pneumoniae is a resistant pathogen involved primarily in hospital-acquired infections. This pathogen is characterized by its notorious resistance to last-line antibiotics, such as carbapenems. Moreover, hypervirulent K. pneumoniae (hvKp) isolates, first identified in Southeast Asia, have emerged globally and are able to cause infections in healthy people. Alarmingly, isolates displaying a convergence phenotype of carbapenem resistance and hypervirulence have been detected in several countries, representing a serious threat to public health. In this work, we analyzed the genomic characteristics of a carbapenem-resistant hvKp isolate recovered in 2022 from a patient with COVID-19 in Chile, representing the first analysis of this type in the country. Our results will provide a baseline for the study of these isolates in Chile, which will support the adoption of local measures aimed at controlling their dissemination.

## OBSERVATION

Klebsiella pneumoniae has emerged as a relevant opportunistic pathogen causing a broad spectrum of infections, including urinary tract infections (UTIs), bloodstream infections, and lung and abdominal cavity infections in hospitalized patients (1). This species is considered among the most common nosocomial pathogens worldwide (2) and belongs to the ESKAPE (Enterococcus faecium, Staphylococcus aureus, Klebsiella pneumoniae, Acinetobacter baumannii, Pseudomonas aeruginosa, and Enterobacter species) group of bacterial organisms, so called due to their ability to evade commonly used antibiotics (3). Accordingly, K. pneumoniae isolates resistant to carbapenems and producing extended-spectrum β-lactamases (ESBLs) have been classified as top-priority pathogens by the World Health Organization (WHO) (4). Globally, infections caused by carbapenem-resistant K. pneumoniae (CR-Kp) isolates have elevated mortality rates (5), highlighting their relevance to human health.

On the other hand, hypervirulent K. pneumoniae (hvKp) isolates have been emerging worldwide recently (6). In this sense, the first hvKp isolate was detected in the mid-1980s in Taiwan and differed from classical K. pneumoniae (cKp) in its ability to generate infections in healthy patients (7). As such, hvKp isolates have been associated with pyogenic liver abscesses, lung abscesses, and metastatic infections, affecting the brain and eyes in healthy adult patients (7). In contrast, cKp isolates are associated with hospital-acquired infections, affecting debilitated patients; hence, hvKp isolates are defined as more virulent than cKp strains (8).

Frequently, cKp isolates possess elevated rates of antibiotic resistance, including to last-line drugs such as third-generation cephalosporins and carbapenems, whereas hvKp isolates are normally susceptible to these drugs (2, 9). Commonly, resistant cKp and hvKp strains belong to different clonal groups (CGs), such as CG15 and CG23, respectively (9). However, there is increasing evidence of the emergence of convergent K. pneumoniae isolates, which are both highly pathogenic and resistant to practically all antibiotics commonly utilized for their treatment (2). This convergence is the result of the mobilization of virulence and resistance genes that are carried on mobile genetic elements, such as plasmids (2). Due to the relevance of the convergence of virulence and resistance in K. pneumoniae, we here present the genomic features of a carbapenem-resistant (CR) hvKp strain (K-2157) recovered in a Chilean hospital.

K. pneumoniae K-2157 was recovered in April 2022 from tracheal aspirate fluid from an intubated patient with coronavirus disease 2019 (COVID-19) in Santiago, Chile. K-2157 was cultured in tryptic soy agar medium and incubated at 37°C for 18 h under aerobic conditions. Antimicrobial susceptibility testing was carried out according to the guidelines of the Clinical and Laboratory Standards Institute (CLSI) (10). The string test was performed on blood agar to identify the hypermucoviscous phenotype (11), while capsular polysaccharide (CPS) quantification was carried out according to Park et al. (12). In addition, we performed the BlueCarba test (13) and conjugation experiments. Virulence biomarkers (*ybtS*, *clbA*, *iucA*, plasmid-borne *rmpA* [_p_*rmpA*], _p_*rmpA2*, K1, and K2) and carbapenemase genes (*bla*_KPC_-like, *bla*_NDM_-like, *bla*_VIM_-like, *bla*_IMP_-like, and *bla*_OXA-48_-like) were screened for using PCR (14, 15).

K-2157 was subjected to whole-genome sequencing (WGS) utilizing the Illumina NextSeq 2000 and Oxford Nanopore GridION sequencing platforms. A Nanopore library was constructed following the SQK-RAD004 protocol (Oxford Nanopore Technologies, Oxford, UK). R9.4.1 flow cells were loaded with 30 μL of DNA from the Nanopore library, and the GridION Mk1 instrument was run for approximately 24 h, until no further sequencing reads could be collected. Fast5 files were base called using Guppy v5.1.12, and output DNA sequence reads with quality values of >8 were saved as Fastq files. A hybrid assembly approach was conducted using Unicycler assembler v0.5.0 (16). Species identification was confirmed using the Tetra Correlation Search (TCS) (https://jspecies.ribohost.com/jspeciesws/#home) with the assembled genome. The genome was analyzed using the Bandage tool (17) to identify the chromosome and plasmid(s). Antibiotic resistance genes (ARGs), sequence type (ST), and virulence factors were screened for using Kleborate v2.2.0 (18). Integrating conjugative elements (ICE) and integrative and mobilizable elements (IME) were detected using the ICE Finder server (https://bioinfo-mml.sjtu.edu.cn/ICEfinder/ICEfinder.html). Those contigs previously identified as plasmids were corroborated using MOB-suite (19), the PlasmidFinder database with ABRicate v1.01.1, and the plasmid database (PLSDB) (20). Seventeen deposited genomes of hvKp isolates collected between 2000 and 2022 were retrieved from GenBank. The inclusion criteria used were to add (i) hvKp from the continent and (ii) genomes of closely related isolates. The genomes were annotated using Prokka v1.14, and the core genome was determined using Roary v3.13.0. Finally, a maximum likelihood (ML) single nucleotide polymorphism (SNP) tree was built using FastTree v2.1.1.

K-2157 was confirmed as K. pneumoniae by the TCS analysis. It displayed resistance to imipenem (IPM), meropenem (MEM), ertapenem (ETP), cefotaxime (CTX), ceftriaxone (CRO), piperacillin-tazobactam (TZP), aztreonam (ATM), and amoxicillin-clavulanic acid (AMC), whereas it was intermediate to ceftazidime (CAZ) and ciprofloxacin (CIP) and susceptible to amikacin (AMK), gentamicin (GEN), colistin (CST), levofloxacin (LEV), and sulfamethoxazole-trimethoprim (SXT). K-2157 was positive for the BlueCarba test, which is congruent with the detection of *bla*_KPC-2_. K-2157 tested positive using the string test in blood agar and showed high CPS production (12). In addition, PCR screening evidenced the presence of virulent biomarkers, revealing that K-2157 exhibits the convergence of carbapenem resistance and a hypervirulent phenotype.

**FIG 1:**
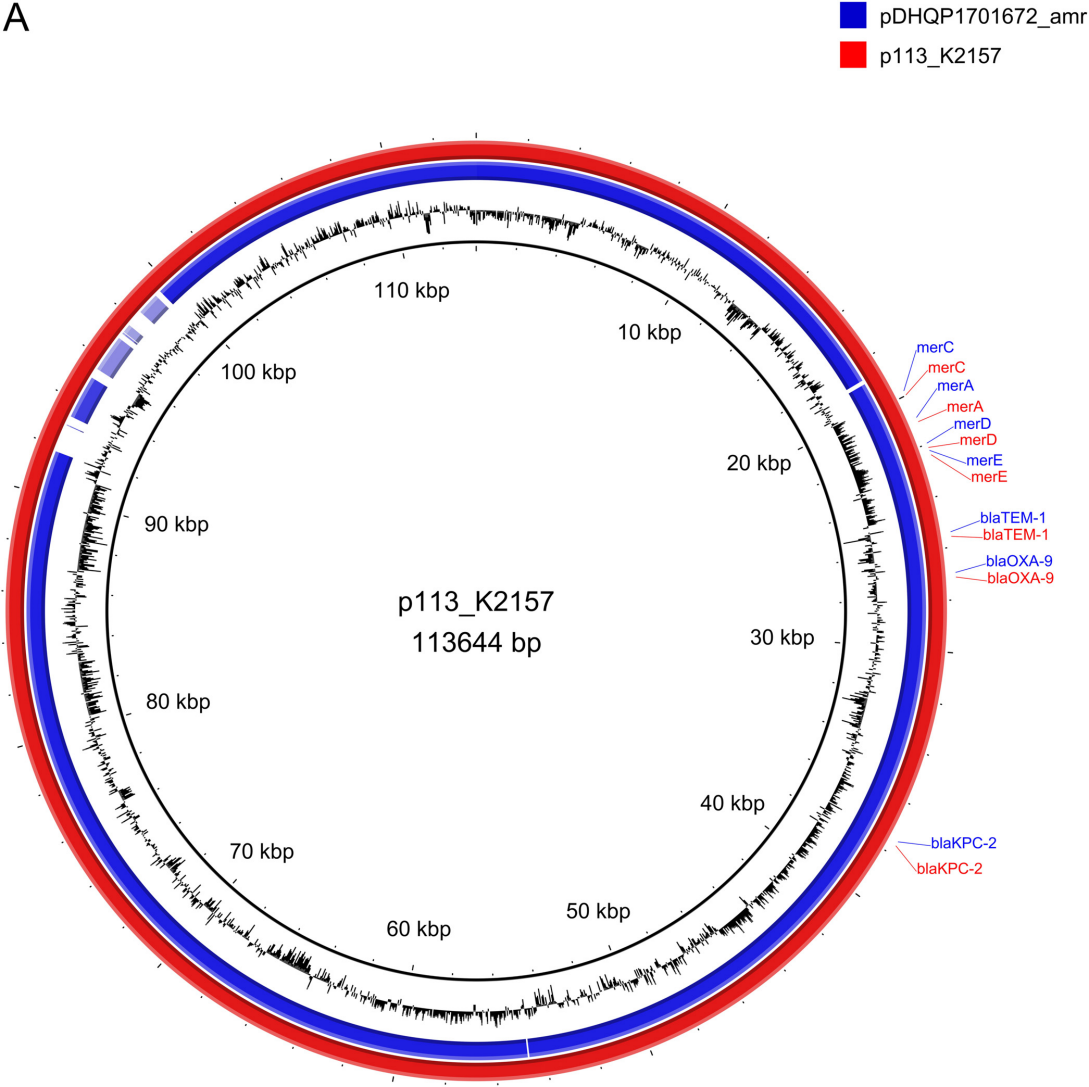

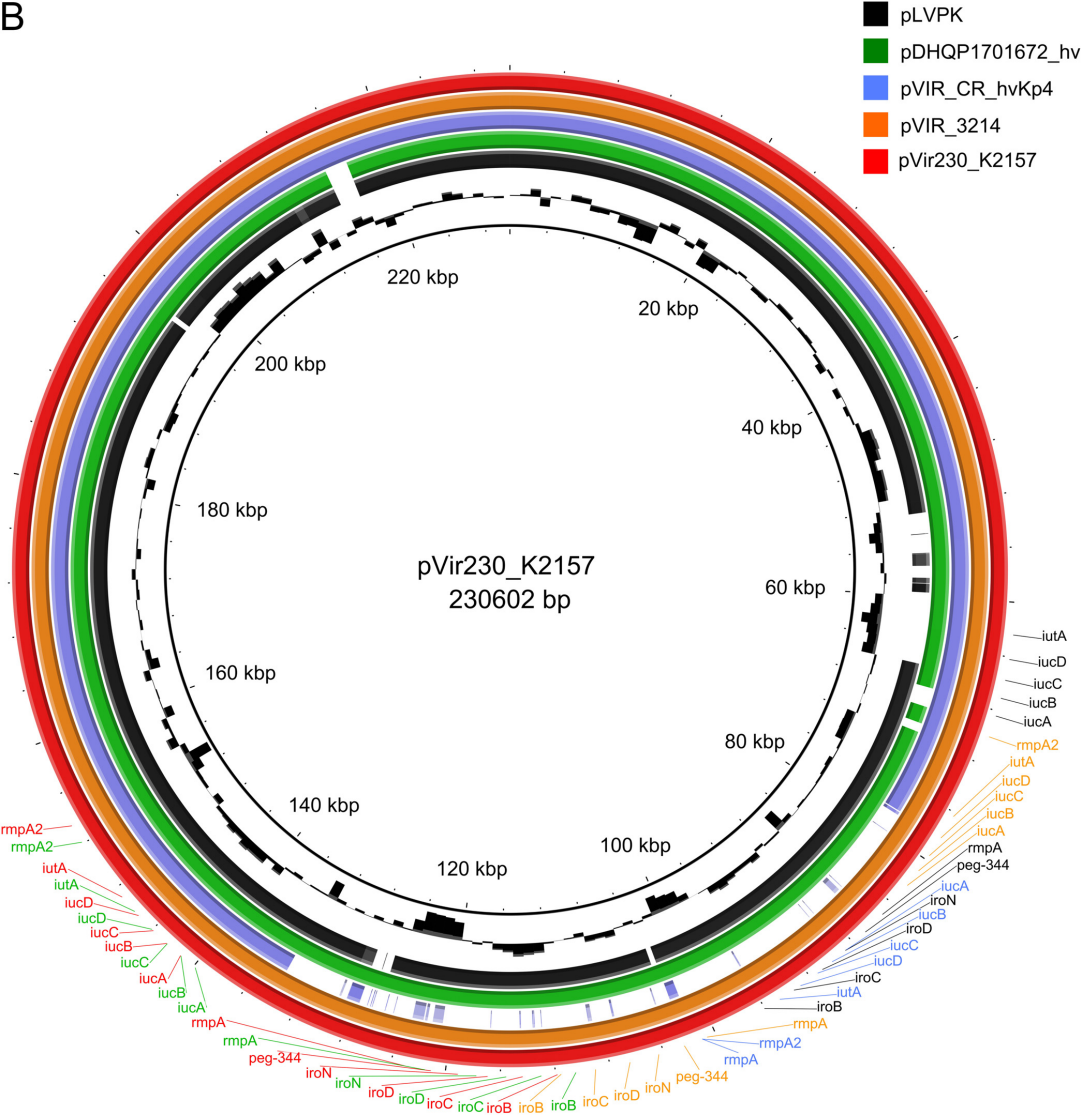
Comparative analysis of resistance plasmid p113_K2157 and virulence plasmid pVir230_K2157 found in strain K-2157 with plasmids deposited in public databases. (A) Resistance plasmids. Plasmid pDHQP1701672_amr (GenBank accession number NZ_CP037744.1) corresponds to the first hypervirulent KPC-2-producing K. pneumoniae isolate collected in the Americas. (B) Virulence plasmids. Plasmid pVIR_3214 (NZ_CP028852.1) matches 100% with pVir230_K2157. Plasmid pVIR_CR_hvKp4 (NZ_MF437313.1) was identified in K. pneumoniae ST11. Plasmid pDHQP (NZ_CP037743.1) was identified in the first hypervirulent KPC-2-producing K. pneumoniae isolate collected in the Americas, and plasmid pLVPK (NC_005249.1) corresponds to the first hypervirulent plasmid described in K. pneumoniae.

The resistome of K-2157 included the *bla*_TEM-1_, *bla*_SHV-190_, *bla*_OXA-9_, *bla*_KPC-2_, *fosA*, *oqxA*, and *oqxB* genes. Furthermore, K-2157 belongs to the ST23 clone associated with the K1 capsular type. Our results are congruent with previous works, since K1-ST23 isolates have been described as an hvKp with moderate virulence in the Galleria mellonella model (21). Classically, the K1-ST23 clone is defined as susceptible to antibiotics, including cephalosporins and carbapenems (18). In this regard, we previously characterized a K1-ST23 hvKp isolate recovered in Chile which was susceptible to carbapenems (22). Since its first description in 2012 in Russia and Germany, this lineage has been identified in several European countries (21, 23). In Europe, the emergence of the convergence phenomenon of K1-ST23 hvKp isolates with carbapenem resistance is mainly mediated by the acquisition of an IncL plasmid harboring the *bla*_OXA-48_ gene (21). However, hvKp K-2157 presented an IncFIB plasmid of 113,644 bp (p113_K2157) harboring the *bla*_KPC-2_, *bla*_OXA-9_, and *bla*_TEM-1_ genes (Fig. 1A). In this sense, p113_K2157 is similar to a plasmid previously detected in K. pneumoniae recovered in the United States in 2013 (Fig. 1A). Moreover, the *bla*_KPC-2_ gene was carried on a Tn*4401* transposon. In addition to the KPC-containing plasmid, K-2157 has another plasmid of 230,602 bp (pVir230_K2157) (Fig. 1B). This plasmid harbored the siderophores genes *iro*, *iut*, and *iuc*, in addition to the _p_*rmpA*, _p_*rmpA2*, and *peg-344* genes, which are all considered biomarkers for hvKp (24). pVir230_K2157 belongs to the IncHI1B type and is similar to a plasmid identified in a K. pneumoniae isolate recovered in China in 2017 (Fig. 1B). Importantly, conjugation experiments using Escherichia coli J53 as the recipient at 25°C and 37°C were negative. Previously, it was asserted that the convergence of virulence and resistance in this clone is mediated by the presence of a fused single plasmid (25), differing from our results. Additionally, K-2157 presented an ICE of 104,622 bp on its chromosome, which is related to ICE*Kp10*, carrying the yersiniabactins *ybtS*, *ybtX*, *ybtQ*, *ybtP*, *ybtA*, *ybtU*, *ybtT*, *ybtE*, *irp1*, *irp2*, and *fyuA* and the colibactin operon *ClbABCDEFGHIJKLMNOPQR*. These results show that the high risk K1-ST23 clone integrates virulence-related ICE*Kp* on its chromosome and can capture additional virulence and resistance epidemic plasmids, producing the convergent phenotype. It is important to highlight the context of the isolation of K-2157, since it was recovered from a COVID-19 patient. Although the evidence is scarce, there is a report from Italy in which hvKp isolates were collected from 36 COVID-19 patients (26). In this case, the infections produced in these susceptible patients were associated with high mortality rates (26), highlighting the relevance of this pathotype during the pandemic.

**FIG 2:**
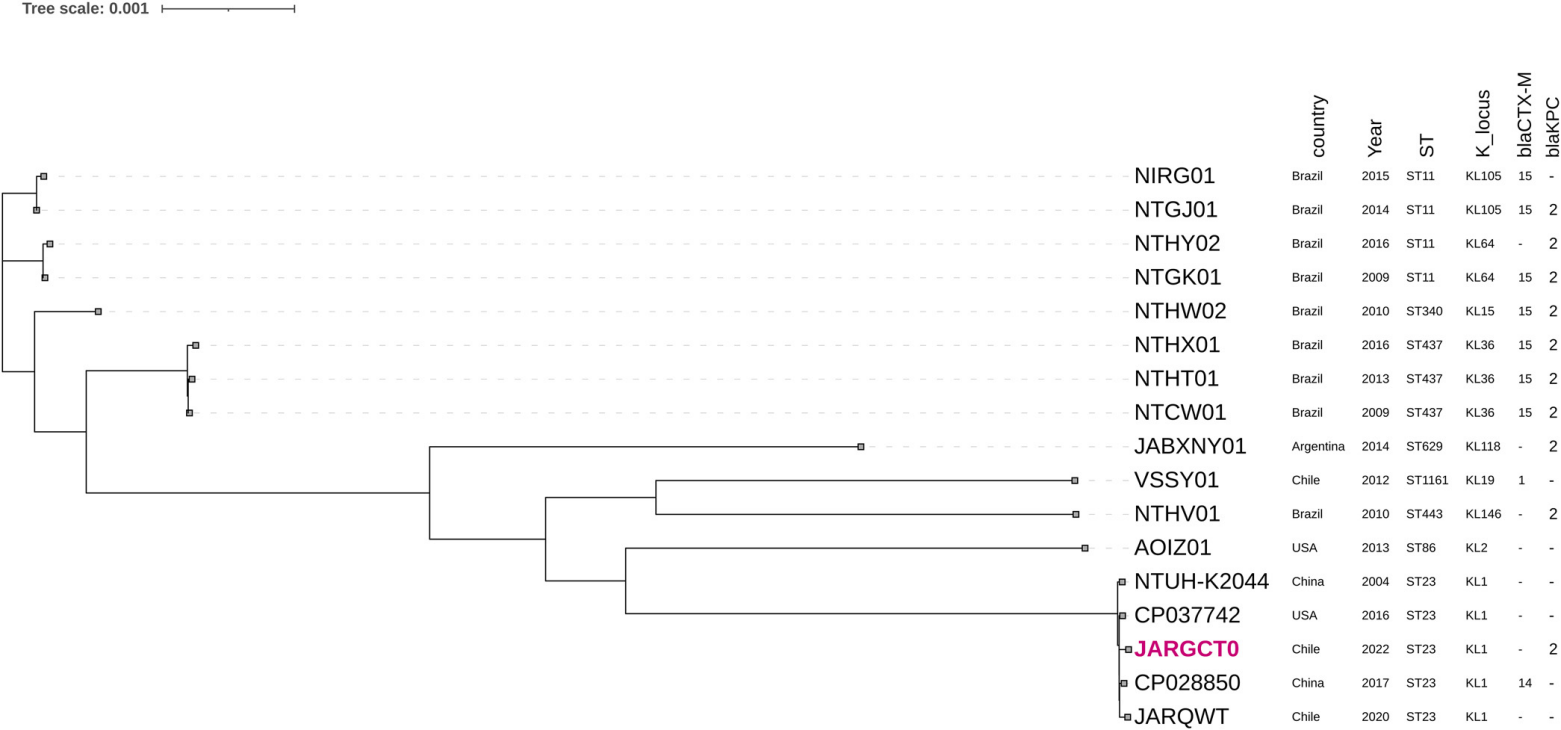
Maximum likelihood phylogenetic tree of the core genomes of 17 hypervirulent K. pneumoniae isolates. The tree was constructed using Roary and FastTree based on core genome SNPs. The country and year of isolation, sequence type (ST), K locus, CTX-M-type extended-spectrum β-lactamases (ESBLs), and KPC carbapenemases are included. The genome of K-2157 is indicated in red.

Moreover, we determined that isolate K-2157 was closely related to hvKp isolates from Chile, China, and the United States (Fig. 2). Interestingly, K-2157 was the only hvKp K1-ST23 isolate containing a carbapenemase gene (*bla*_KPC-2_) (Fig. 2) that determines the emergence of the convergent pathotype. These results suggest that these isolates are circulating in distant geographical areas; therefore, genomic surveillance is paramount to prevent its dissemination, as they possess the potential to generate convergent strains by acquiring a resistance plasmid.

In conclusion, our results represent the first genomic characterization of a hypervirulent and carbapenem-resistant K. pneumoniae isolate from Chile, revealing that this isolate belongs to the virulent K1-ST23 lineage harboring a KPC plasmid. These findings highlight the relevance of performing genomic surveillance to detect strains that represent a serious threat to public health.

### Data availability.

This whole-genome shotgun project has been deposited at DDBJ/ENA/GenBank under the accession number JARGCT000000000. The version described in this paper is version JARGCT010000000.
